# Comparative genomic analysis of a new tellurite-resistant *Psychrobacter* strain isolated from the Antarctic Peninsula

**DOI:** 10.7717/peerj.4402

**Published:** 2018-02-19

**Authors:** Claudia Melissa Muñoz-Villagrán, Katterinne N. Mendez, Fabian Cornejo, Maximiliano Figueroa, Agustina Undabarrena, Eduardo Hugo Morales, Mauricio Arenas-Salinas, Felipe Alejandro Arenas, Eduardo Castro-Nallar, Claudio Christian Vásquez

**Affiliations:** 1 Laboratorio de Microbiología Molecular, Departamento de Biología, Universidad de Santiago de Chile, Santiago, Chile; 2 Departamento de Ciencias Básicas, Facultad de Ciencia, Universidad Santo Tomas Sede Santiago, Santiago, Chile; 3 Center for Bioinformatics and Integrative Biology, Facultad de Ciencias Biológicas, Universidad Andrés Bello, Santiago, Chile; 4 Laboratorio de Microbiología Molecular y Biotecnología Ambiental, Departamento de Química & Centro de Biotecnología Daniel Alkalay Lowitt, Universidad Técnica Federico Santa María, Valparaíso, Chile; 5 Centro de Bioinformática y Simulación Molecular, Universidad de Talca, Talca, Chile

**Keywords:** *Ter* genes, Antarctica, Extremophiles, Tellurite resistance, Phylogenomics

## Abstract

The *Psychrobacter* genus is a cosmopolitan and diverse group of aerobic, cold-adapted, Gram-negative bacteria exhibiting biotechnological potential for low-temperature applications including bioremediation. Here, we present the draft genome sequence of a bacterium from the *Psychrobacter* genus isolated from a sediment sample from King George Island, Antarctica (3,490,622 bp; 18 scaffolds; G + C = 42.76%). Using phylogenetic analysis, biochemical properties and scanning electron microscopy the bacterium was identified as *Psychrobacter glacincola* BNF20, making it the first genome sequence reported for this species. *P. glacincola* BNF20 showed high tellurite (MIC 2.3 mM) and chromate (MIC 6.0 mM) resistance, respectively. Genome-wide nucleotide identity comparisons revealed that *P. glacincola* BNF20 is highly similar (>90%) to other uncharacterized *Psychrobacter* spp. such as JCM18903, JCM18902, and P11F6. Bayesian multi-locus phylogenetic analysis showed that *P. glacincola* BNF20 belongs to a polyphyletic clade with other bacteria isolated from polar regions. A high number of genes related to metal(loid) resistance were found, including tellurite resistance genetic determinants located in two contigs: Contig LIQB01000002.1 exhibited five ter genes, each showing putative promoter sequences (terACDEZ), whereas contig LIQB1000003.2 showed a variant of the terZ gene. Finally, investigating the presence and taxonomic distribution of ter genes in the NCBI’s RefSeq bacterial database (5,398 genomes, as January 2017), revealed that 2,623 (48.59%) genomes showed at least one ter gene. At the family level, most (68.7%) genomes harbored one ter gene and 15.6% exhibited five (including *P. glacincola* BNF20). Overall, our results highlight the diverse nature (genetic and geographic diversity) of the *Psychrobacter* genus, provide insights into potential mechanisms of metal resistance, and exemplify the benefits of sampling remote locations for prospecting new molecular determinants.

## Introduction

The bacterial genus *Psychrobacter* was first described by Juni & Heym (1986) and includes a group of non-motile, oxidase positive, psychrotolerant, Gram-negative rods or coccobacilli isolated from animals and processed foods (Bozal et al., 2003). Bacteria from the *Psychrobacter* genus have also been isolated from natural environments such as Antarctic ornithogenic soils, sea ice, deep-sea, and sea water from the Pacific Ocean and other locations (Bowman, Nichols & McMeekin, 1997; Romanenko et al., 2002). Antarctic isolates belonging to the genus *Psychrobacter* have been described and identified as *P. inmobilis, P. glacincola, P. luti* and *P. fozzi* (Bozal et al., 2003).

The Antarctic territory is the coldest and driest environment on the planet and is exposed to high levels of UV radiation, which favors the production of intracellular Reactive Oxygen Species (ROS) (D’Amico et al., 2006; Potts, 1994). Consequently, microbial communities residing in the Antarctica are unique and some possess diverse strategies to cope with the deleterious effects of ROS and other extreme conditions. In agreement, several microorganisms resistant to antibiotics and other toxicants including tellurite have been isolated from this environment (Arenas et al., 2014; De Souza et al., 2006; Lo Giudice et al., 2013). Many of these organisms can grow at low temperatures and tolerate/resist different compounds, making them ideal candidates for biotechnological applications such as the production of polyunsaturated fatty acids, bioremediation, or as source of industrially useful enzymes (e.g., proteases, lipases) (Brenchley, 1996; Denner et al., 2001).

Tellurite is extremely harmful to most microorganisms, and its toxicity has been associated with the establishment of an oxidative stress status, including ROS generation (Chasteen et al., 2009; Pérez et al., 2007). These ROS are produced as a byproduct of tellurite reduction to its elemental form by either enzymatic or non-enzymatic mechanisms, as visualized by the accumulation of black deposits near the bacterial membrane (Amoozegar et al., 2008; Chasteen & Bentley, 2003; Taylor et al., 1988). Other metal(oid) resistance mechanisms commonly found in bacteria include global cellular responses, cell grouping, uptake control and oxidative stress response, among others (Lemire, Harrison & Turner, 2013).

Some of the genes implicated in tellurite resistance include *trgAB* from *Rhodobacter sphaeroides* (unknown function, encoding likely membrane-associated proteins) (O’Gara, Gomelsky & Kaplan, 1997), *tmp* from *Pseudomonas syringae* (encoding a thiopurine methyltransferase, involved in tellurium alkylation) (Prigent-Combaret et al., 2012), *lpdA* from *Aeromonas caviae* ST (encoding dihydrolipoamide dehydrogenase, involved in tellurite reduction) (Castro et al., 2008) and *gor* from *Pseudomonas* sp. (encoding glutathione reductase, involved in tellurite reduction) (Arenas et al., 2016; Pugin et al., 2014). Tellurite resistance genes were also identified in the *ter* operon (*terZABCDEF*) from *Escherichia coli* and other pathogenic species (Taylor, 1999; Whelan, Sherburne & Taylor, 1997).

Interestingly, the *ter* operon is not only associated with tellurite resistance but also with resistance to bacteriophage infections and to antimicrobial compounds like colicins (Whelan, Colleran & Taylor, 1995). The mechanism of action of the proteins encoded by the *ter* operon remains to be elucidated; however, it is known that they form a multi-subunit complex associated with the inner surface of the bacterial membrane (Anantharaman, Iyer & Aravind, 2012). Likewise, only *terB*, *terC*, *terD* and *terE* have been shown to be directly involved in tellurite resistance (Taylor et al., 2002).

During the Chilean Antarctic expedition ECA-48 in 2012, a bacterium—later identified as *P. glacincola* BNF20—was isolated and characterized; although *P. glacincola* BNF20 was highly resistant to tellurite (MIC 2.3 mM), it did not show increased ROS levels or tellurite reductase activity when exposed to the toxicant (Arenas et al., 2014). In this work, we determined for the first time the genome sequence of a member of the *P. glacincola* species. To gain insight into the potential mechanism(s) of tellurite resistance, we conducted a comparative genomics analysis using available *Psychrobacter* genome sequences. Specifically, we tested if *P. glacincola* BNF20 was phylogenetically related to other *Psychrobacter* Antarctic isolates, and if it harbors *ter* genes. Finally, the *ter* gene taxonomic distribution was assessed using a reference database containing over 5,000 bacterial genomes.

## Materials and Methods

### Strain isolation and culture conditions

*P. glacincola* BNF20 was isolated from a sediment sample collected at King George Island, Antarctica (S62°11′37.6″; W58°56′14.9″) during the ECA-48 Chilean Antarctic Expedition (January 2012). Bacteria were grown at 25 °C as described previously (Arenas et al., 2014) in Lysogenic Broth (LB) medium (Sambrook & Russell, 2001) supplemented with tellurite (200 µg/ml). Strains were identified by sequencing the 16S rRNA gene (accession MF806171) and determining the fatty acid profile. The 16S rRNA gene was sequenced at Pontificia Universidad Católica de Chile using Sanger sequencing with the primers 8F (5′-AGAGTTTGATCCTGGCTCAG-3′) (Turner et al., 1999) and 1492R (5′-ACGGCTACCTTGTTACGACTT-3′) (Lane, 1991). Fatty acid analyses were carried out at DSMZ, Braunschweig, Germany (Kämpfer & Kroppenstedt, 1996). The strain was deposited at DMSZ (Germany), accession # 102806.

To determine the minimal inhibitory concentration (MIC), bacteria were grown overnight in LB medium with shaking at 25 °C. Subsequently, saturated cultures were diluted 1:100 with fresh medium and grown to OD_600_ ∼ 0.4–0.5. Then, 10 µl were added to 990 µl of LB medium containing serial dilutions of defined toxicants in 48-well culture plates. The plates were incubated with constant shaking for 24 h at 25 °C. Assayed toxicants included K_2_TeO_3_, K_2_CrO_4_, CdCl_2_, ZnCl_2_, CuSO_4_, HAuCl_4_, AgNO_3_, NiSO_4_, NaAsO_2_ and Na_2_HAsO_4_.

### 16S rRNA gene phylogenetic analysis

A phylogenetic tree of *P. glacincola* BNF20—based on the partial 16S rRNA gene sequence—was constructed with bootstrap values based on 1,000 replications (Felsenstein, 1985). The nearly complete 16S rRNA gene sequence (1,516 nt) was obtained by merging the PCR sequenced amplicons (accession MF806171) and the sequence obtained by whole genome shotgun sequencing (accession AMK37_RS07000). Sequence alignments, assembly and comparisons, along with best model calculation and construction of the phylogenetic tree were carried out using the MEGA software version 6.0 (Tamura et al., 2013). As outcome, Jukes Cantor model and Pairwise Deletions for gaps treatment was the best fitting model for these sequence data. Nucleotide sequence positions from 16 to 1,535 were considered, according to the *E. coli* K-12 16S rRNA gene sequence numbering (accession AP012306). Scale bar represents 0.01 substitutions per-nucleotide positions. *Moraxella osioensis* DSM 6998^T^ (JN175341) was used as outgroup. The following 16S rRNA sequences from *Psychrobacter* strains were collected from GenBank (accession numbers are given in parentheses): *P. glacincola* DSM 12194T (AJ312213); *P. adeliensis* DSM 15333T (AJ539105); *P. urotivorans* DSM 14009T (AJ609555); *P. arcticus* DSM 17307T (AY444822); *P. cibarius* DSM 16327T (AY639871); *P. cryohalolentis* DSM 17306T (AY660685); *P. frigidicola* DSM 12411T (AJ609556); *P. fozii* NF23T (AJ430827); *P. inmobilis* DSM 7229T (U39399); *P. namhaensis* DSM 16330T (AY722805); *P. aquimaris* DSM 16329T (AY722804); *P. luti* NF11T (AJ430828); *P. alimentarius* DSM 16065T (AY513645); *P. fulvigenes* JCM 15525 (AB438958); *P. piscatorii* JCM 15603 (AB453700); *P. jeotgalli* JCM 11463T (AF441201); *P. arenosus* DSM 15389T (AJ609273) and *P. okhotskensis* JCM 11840 (AB094794).

### Preparation of genomic DNA

*P. glacincola* BNF20 was grown in LB medium at 25 °C for 24 h with constant shaking. DNA was extracted using the Wizard Genomic® DNA Purification Kit (Promega, Madison, WI, USA). The quality and integrity of gDNA was determined by agarose gel (1%) electrophoresis and by determining the 260/280 nm absorbance ratio in a microplate multireader equipment (Tecan Infinite®PRO; Tecan, Männedorf, Switzerland).

### Genome sequencing and annotation

The draft genome sequence of *P. glacincola* BNF20 was determined by a whole-genome shotgun strategy using the Illumina HiSeq 2000 platform with a mate-pair library of 3 kb (Macrogen®). A total of 10.89 million reads were quality filtered and assembled using the A5 pipeline (Tritt et al., 2012). Open reading frame prediction and annotation was carried out using standard operational procedures (Tanenbaum et al., 2010). Gene models were predicted using Glimmer 3.02 (Salzberg et al., 1998) and predicted coding sequences were annotated by comparison with public databases (COG, PFAM, TIGRFAM, UNIPROT, and NR-NCBI). *P. glacincola* BNF20 predicted proteome completeness was assessed by the presence/absence of bacterial orthologs according to the OrthoDB database using BUSCO (Simão et al., 2015). The circular genome map was assembled from *P. glacincola* BNF20 GenBank formatted file (NZ_LIQB00000000.1) using the plotMyGBK wrapper script (https://github.com/microgenomics/plotMyGBK); plotMyGBK uses BioPython and the R platform with the packages rSamTools, OmicCircos, and data.table to produce a vector image of a circular map (Cock et al., 2009; R Development Core Team, 2011; Morgan et al., 2016; Hu et al., 2014; https://github.com/Rdatatable/data.table).

### Nucleotide sequence accession and culture collection number

Raw sequence data from *P. glacincola* BNF20 are available online under the BioProject #PRJNA293364, and Gold ID Gp0145575. The genome project has been deposited at GenBank, accession number NZ_LIQB00000000. The strain was deposited at the DSMZ culture collection, ID number DSM 102806.

### *Psychrobacter* genome dataset

A total of 35 *Psychrobacter* genomes including *P. glacincola* BNF20 were retrieved from NCBI’s Genome and JGI GOLD databases (as of February 2017), where 10 and 24 genomes were annotated to the species and genus level, respectively.

### Phylogenetic relationships and whole-genome nucleotide identity

The average nucleotide identity (ANI) was calculated for the 35-genome dataset using the pyani Python3 module (Pritchard et al., 2016) and the results were visualized using the data.table and pheatmap R packages (https://github.com/Rdatatable/data.table; https://cran.r-project.org/web/packages/pheatmap/index.html). Thirty-one phylogenetic marker genes corresponding to widespread housekeeping genes *dnaG, nusA, rplA, rplD, rplK, rplN, rplT, rpsB, rpsI, rpsM, tsf, frr, pgk, rplB, rplE, rplL, rplP, rpmA, rpsC, rpsJ, rpsS infC, pyrG, rplC, rplF, rplM, rplS, rpoB, rpsE, rpsK, and smpB* were identified in each *Psychrobacter* genome (AMPHORA2; Wu & Eisen, 2008). Each gene was translated under standard genetic code to perform a protein-coding-guided multiple nucleotide sequence alignment, using TranslatorX MUSCLE for the multiple sequence alignment (Abascal, Zardoya & Telford, 2010; Edgar, 2004). Alignments were concatenated using the alignment editor tool Seqotron (Fourment & Holmes, 2016) and the best partition scheme and substitution model was evaluated by PartitionFinder2 (Lanfear et al., 2016). Finally, the software MrBayes v3.2 was used for phylogenetic reconstruction (Ronquist et al., 2012), and the resulting tree was plotted and annotated using FigTree v1.4.3 (http://tree.bio.ed.ac.uk/software/figtree/). Phylogenetic tree annotation was based on the geographic location, according to BioSample database information for each *Psychrobacter* genome.

### Search for metal resistance ortholog genes

To identify metal resistance genes, especially *ter* genes, a bidirectional Blast analysis was performed using the CRB-BLAST method (https://github.com/cboursnell/crb-blast). The BacMet Metal Resistance database (Pal et al., 2014) was used as target and the 35-genome dataset as query. In addition, each genome was re-annotated using the same methodology to identify syntenic genes based on BacMet and Prokka annotation without the bias of different annotation labels as implemented in Prokka v1.12 (Seemann, 2014). Finally, the results were visualized in their genomic context using the in-house script multiGenomicContext (https://github.com/Sanrrone/multiGenomicContext).

### Promoter search

To elucidate if *ter* genes were under the control of one or more promoters, two promoter prediction tools were used on specific contigs where *ter* genes were found (LIQB01000002.1, position 189803-204267; LIQB01000003.2, position 286133-301767): PromPredict algorithm and the online program BPROM (Rangannan & Bansal, 2010; http://www.softberry.com/berry.phtml?topic=bprom&group=programs&subgroup=gfindb). Both results were jointly analyzed.

### Taxonomic classification of *ter***genes**

To investigate if the presence and number of *ter* genes was restricted to certain taxonomic levels, we downloaded all the bacterial reference genomes from the NCBI’s RefSeq database (January 2017; ftp://ftp.ncbi.nlm.nih.gov/refseq/release/bacteria/) and performed a bidirectional Blast searches (crb-blast) against the protein sequences of all *ter* genes in the BactMet database. Then, the in-house script fetchMyLineage (https://github.com/Sanrrone/fetchMyLineage) was employed to obtain the complete lineage of each bacterial genome with at least one *ter* gene match. The results were finally visualized using the R packages: ggplot2, RColorBrewer, devtools, ggjoy purr andreshape2 packages (R Development Core Team, 2011; http://ggplot2.org; https://cran.r-project.org/web/packages/RColorBrewer/index.html; https://github.com/hadley/devtools; https://cran.r-project.org/web/packages/ggjoy/index.html; Wickham, 2007).

## Results

### A new *Psychrobacter* species from the Chilean Antarctic territory

**Figure 1:**
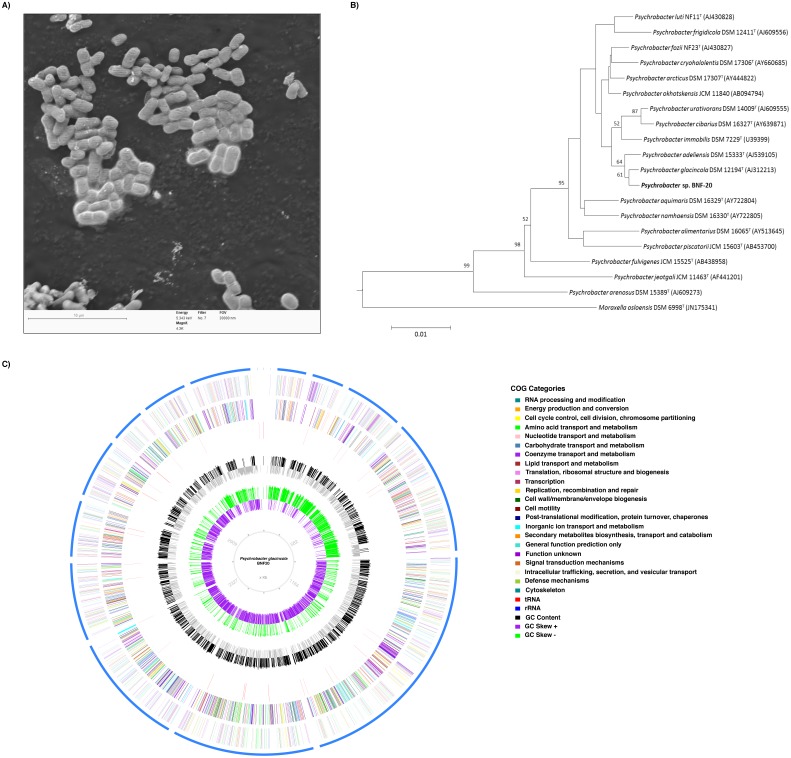
Phylogenetic, morphological and genomic characteristics of *P. glacincola* BNF20. (A) Scanning electron micrograph showing the morphology and dimensions of *P. glacincola* BNF20. Samples were stained with uranyl acetate (0.5% w/v) and examined using a low-voltage electron microscope (Delong Instruments, LVEM5), with a nominal operating voltage of 5 kV. Bar represents 10 µm. (B) Phylogenetic tree of *P. glacincola* BNF20 based on the partial 16S rRNA gene sequence (Accession number MF806171). *Psychrobacter* ingroup was rooted using *Moraxella osloensis* DSM 6978T as outgroup. (C) Circular map of the 18-scaffold draft genome with coding sequences colored by COG categories. Inner circles represent GC Skew and GC content.

*P. glacincola* BNF20 was isolated from Antarctic sediments and is a Gram-negative, non-motile, aerobic, oxidase positive, rod-shaped bacterium with an average dimension of 1.66 µm length and 1.09 µm width (Table 1, Fig. 1A). The fatty acid composition was determined at the DSMZ Institute (Germany) and showed that the major fatty acid was cis-9 octadecenoic acid C_18:1_ *ω*9c (63.78%). The morphology description and major fatty acid component agrees with previous studies of Antarctic *Psychrobacter* isolates (Bozal et al., 2003). Initially, BNF20 was erroneously identified as *P*. *inmobilis*, based on a partial 16S rRNA gene sequence (Arenas et al., 2014). However, re-sequencing and a phylogenetic analysis of the partial 16S rRNA gene revealed that it is related to the *P. glacincola* species, family *Pseudomonadaceae* from the Gammaproteobacteria class (Fig. 1B). Altogether, morphology (electron microscopy), biochemical properties, partial (Sanger) and full length (NGS) 16 S rRNA gene sequence analysis, and fatty acid composition suggest that isolate BNF20 is member of the *P. glacincola* species.

**Table 1:** Classification and general features of *P. glacincola* BNF20.

**MIGS ID**	**Property**	**Term**
	Classification	Domain: *Bacteria*
		Phylum: *Proteobacteria*
		Class: *Gammaproteobacteria*
		Order: *Pseudomonadales*
		Family: *Moraxellaceae*
		Genus: *Pychrobacter*
		Species: *Psychrobacter glacincola*
		(Type) strain: BNF20
	Gram stain	Negative
	Cell shape	Rod
	Motility	Non-Motile
	Sporulation	Non-sporulating
	Temperature range	Psychrotolerant
	Optimum temperature	25 °C
	pH range; Optimum	Not tested; 7.4
	Carbon source	Citrate, acetate, pyruvate
MIGS-6	Habitat	Antarctic sediment
MIGS-6.3	Salinity	0–10% NaCl (w/v)
MIGS-22	Oxygen requirement	Aerobic
MIGS-15	Biotic relationship	Free-living
MIGS-14	Pathogenicity	Potentially pathogenic
MIGS-4	Geographic location	King George Island, Antarctica
MIGS-5	Sample collection	January, 2012
MIGS-4.1	Latitude	62°11′S
MIGS-4.2	Longitude	58°56′W
MIGS-4.4	Altitude	Not registered

### *P. glacincola* BNF20 tolerates high tellurite and chromate concentrations

Several tests were carried out to determine if BNF20 was resistant to multiple metals. Besides tellurite (used in the initial selection), *P. glacincola* BNF20 was 4 times more resistant to chromate than the sensitive strain *E. coli* BW25113 under optimal growth conditions (Table 2). However, *P. glacincola* BNF20 growth was impaired in the presence of all other metal(loid)s tested, including Cu^2+^, Cd^2+^, Hg^2+^, Zn^2+^, ${\mathrm{AuCl}}_{4}^{1-}$, Ni^2+^, ${\mathrm{AsO}}_{4}^{2-}$, ${\mathrm{AsO}}_{3}^{1-}$, and Ag^1+^.

**Table 2:** Minimal inhibitory concentrations (mM) of different metal(loid)s for *P. glacincola* BNF20, and *E. coli* BW25113 (reference).

Metal	BNF20	BW25113
${\mathrm{TeO}}_{3}^{2-}$	2.3	0.004
Cu^2+^	3.12	6
Cd^2+^	0.062	1
Hg^2+^	0.0039	0.01
Zn^2+^	0.5	2
${\mathrm{CrO}}_{4}^{2-}$	6	1.5
${\mathrm{AuCl}}_{4}^{1-}$	0.015	0.16
Ni^2+^	1.25	5
${\mathrm{AsO}}_{4}^{2-}$	40	80
${\mathrm{AsO}}_{2}^{1-}$	5	10
Ag^1+^	0.015	0.063

### First draft genome of *Psychrobacter glacincola* BNF20

Previous studies showed that *P. glacincola* BNF20 was highly resistant to tellurite (MIC ∼2.3 mM, Arenas et al., 2014). Although tellurite reduction is often accompanied by the formation of black deposits of elemental tellurium in resistant organisms, this phenotype was not observed in *P. glacincola* BNF20. To further investigate the mechanism(s) of tellurite resistance in *P. glacincola* BNF20, we sequenced the whole genome in search for genetic determinants implicated in metal(loid) resistance. The assembled genome of *P. glacincola* BNF20 consisted of 3,490,622 bp, 18 scaffolds, with an average G + C content of 42.76% (Fig. 1C; NCBI Reference Sequence: NZ_LIQB00000000.1). The predicted proteome scored 100% completeness according to the presence of highly conserved ortholog genes in bacteria (BUSCO analysis). A set of 47 tRNA genes and one copy of the rRNA operon were also identified. From a total of 2,968 predicted CDS, 2,872 (96.7%) ORFs matched coding sequences available in public databases, of which 2,515 were assigned (84.7%) or not (352 CDS, 19.31%) to COG categories (NZ_LIQB00000000.1).

### *P. glacincola* BNF20 is evolutionarily divergent from other Antarctic *Psychrobacter***isolates**

**Figure 2:**
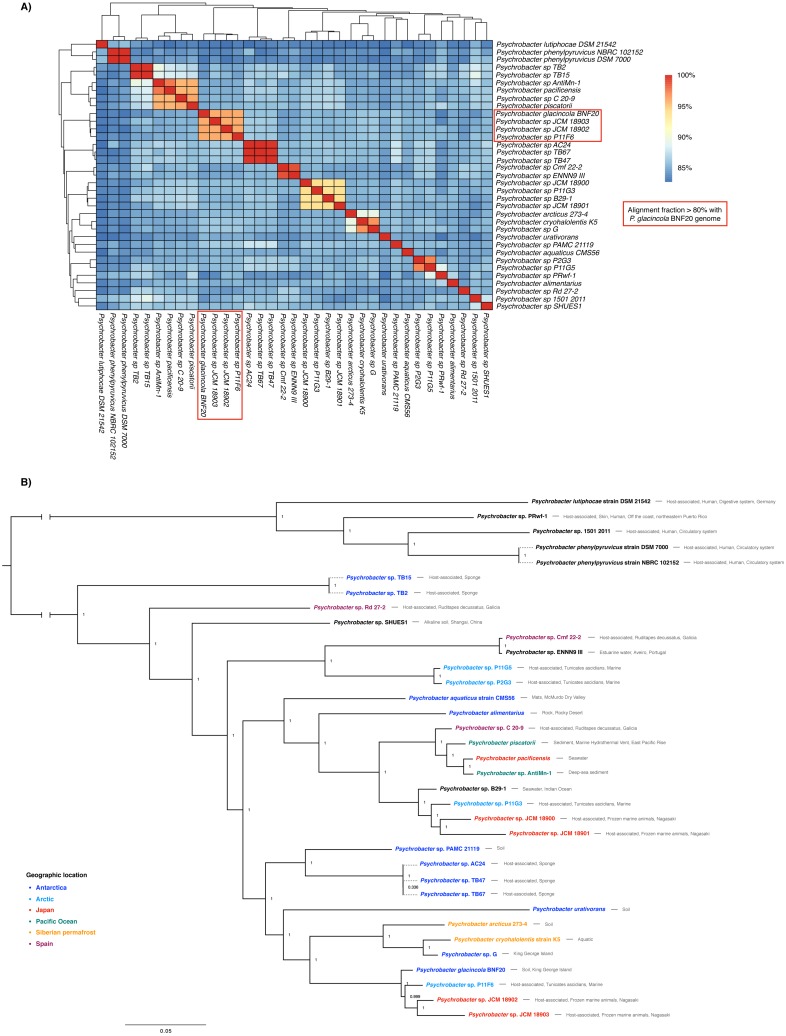
Whole genome nucleotide identity and multi-locus phylogenetic analysis. (A) Average nucleotide identity (ANI) in the 35-genome *Psychrobacter* dataset. *P. glacincola* BNF20 forms a cluster with other three *Psychrobacter* genomes with an alignment fraction over 80%. (B) Bayesian multi-locus phylogenetic analysis of the genomic sequences from the indicated *Psychrobacter* members. Taxa are colored by geographic location. Node values correspond to posterior probabilities, and the phylogeny was mid-point rooted.

The genome sequence of *P. glacincola* BNF20 was compared to other 34 available genomic sequences by estimating ANI values and performing a multi-locus phylogenetic analysis (Fig. 2). Besides *P. glacincola* BNF20, the full dataset was composed of 10 named and 24 unnamed *Psychrobacter* species, respectively. *P. glacincola* BNF20 exhibited an average nucleotide identity >95% and an alignment fraction of over 80% with 3 isolates designated as *Psychrobacter* sp*.* JCM18903 (GCA_000586475.1), *Psychrobacter* sp. JCM 18902 (GCA_000586455.1) (Kudo et al., 2014) and *Psychrobacter* sp. P11F6 (GCA_001435295.1) (Moghadam et al., 2016), of which none was isolated from Antarctica. We did not find any genome comparison against BNF20 of >96.5% ANI and >60% alignment fraction, which has been suggested as a “genomic boundary” for bacterial species (Fig. 2A; Varghese et al., 2015). While some of the available genomes come from Antarctic isolates, none of them showed high ANI values (>90%): *P. aquaticus* (85%; GCA_000471625.1); *P. alimentarius* (85%; GCA_001606025.1); *P. urativorans* (85%; GCA_001298525.1); TB15 (84%, GCA_000511655.1), G (86%, GCA_000418305.1); PAMC 21119 (86%, GCA_000247495.2); TB2 (84%, GCA_000508345.1); TB47 (86%, GCA_000511045.1); TB67 (86%, GCA_000511065.1) and AC24 (86%, GCA_000511635.1).

Supporting our previous results, multi-locus phylogenetic analysis showed that *P. glacincola* BNF20 is more related to P11F6 (isolated from Tunicate ascidians from the Arctic, Moghadam et al., 2016), JCM 18902 and 18903 (isolated from frozen porpoise *Neophocaena phocaenoides,* Kudo et al., 2014). Antarctic isolates PAMC21119 and G (from King George Island, Moghadam et al., 2016; Che et al., 2013) belong to a polyphyletic group and do not form a monophyletic clade with *P. glacincola* BNF20, highlighting the heterogeneous nature of the *Psychrobacter* genus (Fig. 2B). All nodes of the phylogeny were well supported (posterior probability >  0.99).

### *P. glacincola* BNF20 encodes multiple metal resistance determinants

As *P. glacincola* BNF20 was isolated from King George Island sediments, a place where heavy metal contamination has not been previously reported, we searched for genes known to be involved in metal resistance that could explain the observed tellurite and chromate resistance of strain BNF20 (BacMet database; Pal et al., 2014). Type and gene copy number distribution was not uniform in the 35-genome *Psychrobacter* dataset (Table S3).

**Figure 3:**
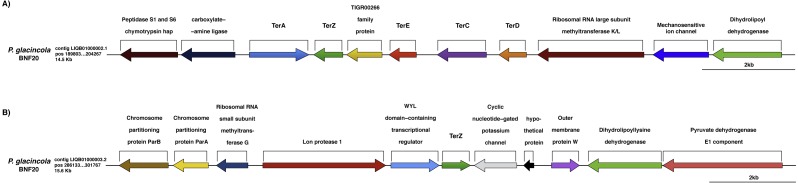
Genomic context of the *ter* genes harbored by *P. glacincola* BNF20. (A) Context of the gene cluster located at nucleotide (nt) positions 189,803-204,267 of the Contig LIQB1000002.1. (B) Contig LIQB01000003.2, located at nt positions 286,133–301,767 exhibits an extra copy of the *ter*Z gene.

Specifically, ∼100 genes possibly conferring metal resistance were identified in the genome of *P. glacincola* BNF20, of which some are related to chromate resistance, including *chrL* (BAC0361; regulatory protein, involved in chromate resistance), *chrR* (BAC0538; chromate reductase), *mdrL/yfmO* (BAC0209; multidrug efflux protein *yfmO*) and *ruvB* (BAC0355; ATP-dependent DNA helicase), and some to tellurite resistance—the so-called *ter* genes (Whelan & Colleran, 1992), including *terA* (BAC0386), *terC* (BAC0388), *terD* (BAC0389), *terE* (BAC0390) and *terZ* (BAC0392) (Fig. 3, Table S3). Two other genes apparently involved in tellurite resistance, *ruvB* (BAC0355; ATP-dependent DNA helicase) and *pitA* (BAC0312; low-affinity inorganic phosphate transporter 1), were also identified.

### Organization of *ter* genes in *P. glacincola* BNF20

Given that (i) tellurite is by far more toxic for bacteria than other metals (Taylor, 1999) and (ii) it is scarce in the Earth’s crust (Turner, Borghese & Zannoni, 2012), finding tellurite resistance determinants in *P. glacincola* BNF20 was somewhat unexpected. Since to date the presence of *ter* genes in Antarctic microorganisms has not been reported, we focused the following analyses our study on them.

The *ter* genes were originally described as part of an *E. coli* operon exhibiting the *terZABCDE* structure (Taylor et al., 2002). *P. glacincola* BNF20 harbors *terA*, *terZ*, *terE*, *terC* and *terD* orthologs, but not *terB* (Fig. 3A); *terA* shows the opposite transcriptional orientation than the rest of the *ter* genes, while *terZ* is duplicated and is contained in different contigs (Fig. 3B). In addition, the expression of all *ter* genes in *P. glacincola* BNF20 seems to be regulated by individual promoters (PromPredict and BPROM analyses), suggesting that they are organized as a gene cluster rather than as an operon.

Three members of the *Psychrobacter* genus contained one *ter* gene (*P. phenylpyruvicus* (*terZ*, GCA_000685805.1), *P. lutiphocae* (*terZ*, GCA_000382145.1) and *P. sp.* ENNN9 III (*terD*, GCA001462175.1)), while the rest had different combinations of them (Fig. S1).

In *P. glacincola* BNF20, the context of the *ter* gene cluster is similar to other isolates like *Psychrobacter* sp. JC18902 (GCA_00058655.1), *Psychrobacter* sp. G (GCA_000418305.1), *Psychrobacter* sp. TB67 (GCA_000511065.1), *Psychrobacter* sp. AC24 (GCA_000511635.1), *Psychrobacter* sp. TB47 (GCA_00051045.1) and *P. arcticus* 273-4 (GCA_000012305.1). Interestingly, in all analyzed *Psychrobacter* genomes the *ter* gene cluster also contains a gene encoding a protein of the TIGR00266 family (unknown function, Fig. S1).

### *ter* genes are distributed over several bacterial Phyla

**Figure 4:**
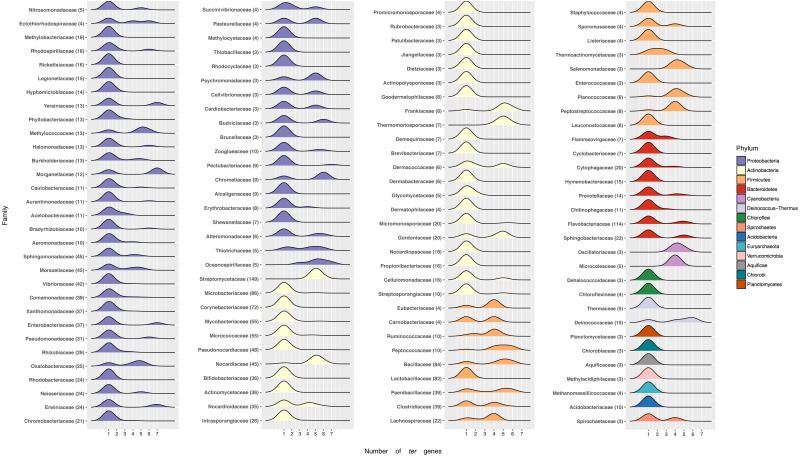
Number of *ter* genes in Bacterial families. Distribution of *ter* genes present in the indicated phyla. Only taxonomic classifications (Phylum and Family) with at least three bacterial genomes encoding at least one *ter* gene are shown.

To determine the frequency of *ter* genes in known bacterial genomes, their taxonomic distribution was evaluated. In general, *ter* genes are more commonly found in Gram-positive than in Gram-negative bacteria. Using NCBI’s RefSeq database (>5,000 genomes; accessed January 2017), we found that 48.59% of them contained *ter* genes (26 out of 30 bacterial Phyla). While, at the genus level, most genomes had one *ter* gene (67.95%) (Table S4, Fig. 4), others harbor two (2.31%), three (0.69%), four (5.24%), six (4.61%) or seven (1.15%) *ter* genes. Interestingly, the second most abundant combination of *ter* genes in genomes was five (18.04%), which could suggest evolutionary constrains.

At the phylum level most Proteobacteria contain one *ter* gene, with a few exceptions showing up to 7, including *Yersiniacee, Morganellaceae, Enterobacteriaceae* and *Erwiniaceae*. A similar pattern is observed in other Phyla, except for Firmicutes where genomes exhibit a defined array of *ter* genes (Fig. 4). Interestingly, while members belonging to the best represented family in RefSeq, i.e., *Streptomycetaceae* (149 genomes) exhibit five or six *ter* genes, in other well-represented families such as *Flavobactericidae* only 26 out of 114 genomes exhibit five *ter* genes (23%). Within the *Moraxelaceae* family, nine out of 45 genomes show five *ter* genes (20%, including BNF20), which agrees with the complete family database distribution (∼18% with 5 *ter* genes).

## Discussion

Here we show for the first time the genome sequence of a *P. glacincola* species isolated from Antarctica, which can tolerate high concentrations of tellurite and chromate. *P.  glacincola* BNF20 showed to be 4- and 500-fold more resistance to chromate and the tellurium oxyanion tellurite than *E. coli* BW25113 (Table 2).

Previous studies showed that defined toxicants can trigger common responses or repair mechanisms (Miranda et al., 2005), suggesting that tellurite and chromate resistance could be related. Besides tellurite and chromate, *P. glacincola* BNF20 genome encodes resistance determinants associated to a number of other heavy metal(loid)s such as arsenic, cadmium, copper and mercury (Table S3). Interestingly, tellurite resistance in *P. glacincola* BNF20 did not correlate with a strong tellurite reduction, as previously reported (Arenas et al., 2014), which prompted us to search for genes associated with tellurite resistance in its genome. Identifying these genetic resistance determinants could be useful as the *Psychrobacter* genus has been proposed as good candidate for biotechnological applications including bioremediation (Lasa & Romalde, 2017).

Members of the *Psychrobacter* genus are versatile and have been isolated from different places with low temperatures—including Antarctica—as well as from some animal hosts including skin, fish gills and guts and human blood, among others (Bowman, Nichols & McMeekin, 1997; Bozal et al., 2003; Romanenko et al., 2002). However, isolates from similar environments show high genomic variability, as evidenced by ANI analysis (Table S2). A multi-locus phylogenetic analysis revealed that Antarctic *Psychrobacter* isolates do not form a monophyletic group (Fig. 2). In this context, the presence of *ter* genes is correlated to some extent with their genomic structure. In fact, higher ANI values reflected a more similar *ter* gene organization. Thus, *P. glacincola* BNF20 exhibited a very close *ter* gene organization with the three closest members *Psychrobacter* sp. P11F6, JCM18902 and JCM18903 (Fig. 2, Fig. S1).

Psychrophilic and psychrotolerant microorganisms require several genes to increase their phenotypic flexibility to survive in extreme environments such as cold habitats. Thus, in addition to genes associated with cold shock proteins, membrane fluidity, among others, the presence of metal(loid) resistance genes seems to favor their adaptation (Dziewit & Bartosik, 2014; Rodríguez-Rojas et al., 2016). This is also the case of *P.  glacincola* BNF20, which harbors over 100 putative metal resistance genes (Table S2). In principle and even though this high number of genes predicted bacterial resistance to a number of metal(loid)s, MIC determinations showed that *P. glacincola* BNF20 was only resistant to chromate and tellurite (MIC 6 and 2.3 mM, respectively). Chromate resistance genes included *chrI* (regulatory protein of *Ralstonia metallidurans* CH34; Juhnke et al., 2002), *chrR* (encoding a chromate reductase; Park et al., 2000), *mdrL/yfmO* (multidrug efflux transporter in *Listeria monocytogenes*; Mata, Baquero & Pérez-Díaz, 2000) and *ruvB*, encoding a DNA helicase involved in both chromate and tellurite resistance in *P. aeruginosa* PAO1 (Miranda et al., 2005). Genes related to tellurite resistance identified in *P. glacincola* BNF20 included the phosphate transporter *pitA* (Elías et al., 2012) and a cluster of *ter* genes (Whelan, Colleran & Taylor, 1995), composed of *terA, terZ, terC, terD and terE*, which exhibit a different organization as compared to other *ter* gene clusters previously described (Fig. 3). Although *ter* refers to tellurite resistance, the same genes participate in resistance to phages, colicins (Whelan, Sherburne & Taylor, 1997) and to other oxidative stress-generating antimicrobials (Taylor, 1999), which could be the result of transcriptional control by a common regulator, OxyR (Ni et al., 2014).

A number of reasons may explain the observed discordances among MIC values (i.e., Hg, Cu, As, etc.) and the respective resistance genes identified in this bacterium. For instance, *P. glacincola* BNF20 sensitivity to mercury could be a result of the absence of some genes (i.e., *mer*T) belonging to the *mer* operon, which could render it non-functional (Boyd & Barkay, 2012). Similarly, the absence of the c*us*S gene (Cu sensor) in the *P. glacincola* BNF20 genome could be responsible for its copper sensitivity, in spite the presence of other genes that participate in Cu homeostasis (Rensing & Grass, 2003).

Tellurite resistance-associated *ter* genes are grouped in three different families: (i) TerC, encompassing transmembrane proteins, (ii) TerD, which includes the cytoplasmatic paralogs TerD, TerA, TerE, TerF and TerZ (Anantharaman, Iyer & Aravind, 2012), and (iii) TerB, representing proteins that are directly associated with the inner surface of the cell membrane, although they also have a cytoplasmatic localization (Alekhina, Valkovicova & Turna, 2011). As mentioned, TerC interacts with TerD, TerB and other proteins showing different cell functions (Turkovicova et al., 2016).

Most bacteria carrying *ter* genes display a similar transcriptional organization. Thus *terZABCDEF*, *terZABCDE* and *TerABD* present in *E. coli* O157:H7, *Proteus* sp. and *D. radiodurans*, respectively, are operons (Makarova et al., 2001; Toptchieva et al., 2003; Taylor et al., 2002). The *Psychrobacter* genus represents an exception to this rule, with *terA* lying in the opposite transcriptional orientation (Fig. S1).

Transcriptomic and proteomic assays have shown that *terB* is expressed when *E. coli* or *D. radiodurans* are exposed to tellurite (Anaganti et al., 2015; Taylor et al., 2002). TerB seems to be essential for tellurite resistance and interacts with some cytoplasmatic proteins such as the alpha subunit of ATP synthase, G subunit of the NADH-dependent quinone oxidoreductase and DnaK chaperone, among others (Alekhina, Valkovicova & Turna, 2011). Given that *P. glacincola* BNF20 lacks *terB*, we hypothesize that there must be another gene product that mediates tellurite resistance.

Based on their genetic background, *ter* genes have also been classified into different groups (I–IV) (Anantharaman, Iyer & Aravind, 2012). In this context and given its similitude with the *ter* genes found in *Psychrobacter* sp. PRwf-1, *P. glacincola* BNF20 would belong to group I, which contains a gene encoding a protein exhibiting the AIM24 domain, also found in the *P. glacincola* BNF20 TIGR00266 protein. Although no role has been ascribed to it in prokaryotes, in higher organisms it is an internal membrane protein related to mitochondrial biogenesis which is required for yeast respiration (Deckers et al., 2014). The AIM24 domain exhibits a double beta-helix folding, which is frequently found in genes neighboring TerD, suggesting that both proteins could interact (Anantharaman, Iyer & Aravind, 2012).

Deciphering the origins of bacterial operons is not straightforward, and there are some hypotheses that try to explain their formation. An interesting example is the piecewise model, which states that the *his* operon *(hisGDCBHAFIE)* was gradually formed. Phylogenetic analyses of the Proteobacterial phylum *his* genes showed their progressive grouping, which suggests that they were located in nearby zones of the chromosome in closely related microorganisms. Following, new events ended with the formation of the *hisBHAF* central core and the whole operon (Fani, Brilli & Liò, 2005). A future hypothesis to test is whether the *ter* operon has a similar evolutionary origin.

To evaluate the taxonomical distribution of *ter* genes in the Bacterial kingdom, the 5,398 genomes retrieved from the NCBI’s RefSeq bacterial database were screened. About 48.6% of them (2,623 genomes) were found to contain *ter* genes. While at the family level most (68.7%) harbored one *ter* gene (chiefly *terC*) and 15.6% exhibited five (including *P. glacincola* BNF20), at the class level the number of genomes exhibiting at least one *ter* gene was *Gammaproteobacteria* (379), *Alphaproteobacteria* (253) and *Bacilli* (247). Finally and regarding phyla, Proteobacteria, Actinobacteria and Firmicutes had 867, 854 and 361 genomes containing at least one *ter* gene, respectively (Fig. 4, Table S4).

Within the Proteobacteria phylum, most families had only one *ter* gene, while others up to 7 (Morganellaceae, Yersiniaceae), 6 (Chromatiacceae, Budviciaceae), 5 (Moraxellceae, Burkholderiaceae), 4 (Erythrobacteraceae), etc. (Fig. 4). In this context, it would be interesting to carry out phylogenetic analyses to understand the evolution of these *ter* genes and how the currently known *terZABCDEF* operon was formed (Taylor, 1999; Whelan, Sherburne & Taylor, 1997).

Finally, it was found that—in general—Gram-positive microorganisms contain more *ter* genes than Gram-negative bacteria (Table S4). This is interesting because it is generally accepted that they also show higher tellurite resistance (Taylor, 1999). For instance, *Streptomyces* and *Bacillus* genera comprise 137 and 65 genomes carrying up to 5–6 *ter* genes, respectively, suggesting that *ter* gene copy number could be related to the high resistance to tellurite observed in *S. coelicolor* and *Geobacillus stearothermophilus* (Moscoso et al., 1998; Sanssouci et al., 2011).

## Conclusions

A new species of Antarctic bacteria exhibiting high tellurite resistance was isolated and identified as *P. glacincola* BNF20. Although within the genus the percent of sequence coverage is low, its genomic sequence is similar to other uncharacterized genomes and contains a large number of genes implicated in metal(loid) resistance, especially chromate and tellurite. The transcriptional orientation of tellurite resistance (*ter*) genes in *P. glacincola* BNF20 is different to that described in other microorganisms and most likely do not function as an operon. The wide distribution of *ter* genes in the bacterial world suggests that they play an important physiological role.

##  Supplemental Information

10.7717/peerj.4402/supp-1 Table S1 *Psychrobacter* genome dataset used in this study

10.7717/peerj.4402/supp-2 Table S2 Average nucleotide identity (ANI) and per cent of alignment fraction among the indicated *Psychrobacter* species

10.7717/peerj.4402/supp-3 Table S3 Metal(loid) resistance genes in the indicated *Psychrobacter* species

10.7717/peerj.4402/supp-4 Table S4 Per cent of bacterial genomes exhibiting *ter* genes

10.7717/peerj.4402/supp-5 Figure S1 Genetic context of *ter* genes in the *Psyc* h*robacter* genus

## Additional Information and Declarations

### Data Availability

The following information was supplied regarding data availability: The genome sequence described here is accessible via GenBank accession number NZ_LIQB00000000.1. The raw reads and genome assembly are publicly available in NCBI under Bioproject (NCBI) accession number PRJNA293364.

## References

[ref-1] Abascal F, Zardoya R, Telford MJ (2010). TranslatorX: multiple alignments of nucleotide sequences guided by amino acid translations. Nucleic Acids Research.

[ref-2] Alekhina O, Valkovicova L, Turna J (2011). Study of membrane attachement and *in vivo* co-localization of TerB protein from uropathogenic *Escherichia coli* KL53. General Physiology and Biophysics.

[ref-3] Amoozegar MA, Ashengroph M, Malekzadeh F, Razavi MR, Naddaf S, Kabiri M (2008). Isolation and initial characterization of the tellurite reducing moderately halophilic bacterium, *Salinicoccus* sp. strain QW6. Microbiological Research.

[ref-4] Anaganti N, Basu B, Gupta A, Joseph D, Apte SK (2015). Depletion of reduction potential and key energy generation metabolic enzymes underlies tellurite toxicity in *Deinococcus radiodurans*. Proteomics.

[ref-5] Anantharaman V, Iyer LM, Aravind L (2012). Ter-dependent stress response systems: novel pathways related to metal sensing, production of a nucleoside-like metabolite, and DNA-processing. Molecular BioSystems.

[ref-6] Arenas F, Pugin B, Henríquez N, Arenas M, Díaz W, Pozo F, Muñoz CM, Chasteen TG, Pérez-Donoso JM, Vásquez CC (2014). Isolation, identification and characterization of highly tellurite-resistant, tellurite-reducing bacteria from Antarctica. Polar Science.

[ref-7] Arenas M, Vargas-Pérez J, Morales W, Pinto C, Muñoz Díaz P, Cornejo F, Pugin B, Sandoval JM, Díaz-Vásquez WA, Muñoz Villagrán C, Rodríguez-Rojas F, Morales EH, Vásquez CC, Arenas FA (2016). Flavoprotein-mediated tellurite reduction: structural basis and applications to the synthesis of tellurium-containing nanostructures. Frontiers in Microbiology.

[ref-8] Bowman JP, Nichols DS, McMeekin TA (1997). *Psychrobacter glacincola* sp. nov., a halotolerant, psychrophilic bacterium isolated from Antarctic sea. Systematic and Applied Microbiology.

[ref-9] Boyd ES, Barkay T (2012). The mercury resistance operon: from an origin in a geothermal environment to an efficient detoxification machine. Frontiers in Microbiology.

[ref-10] Bozal N, Montes MJ, Tudela E, Guinea J (2003). Characterization of several *Psychrobacter* strains isolated from Antarctic environments and description of *Psychrobacter luti* sp. nov. and *Psychrobacter fozii* sp. nov. International Journal of Systemaic and Evolutionary Microbiology.

[ref-11] Brenchley JE (1996). Psychrophilic microorganisms and their cold-active enzymes. Journal of Industrial Microbiology.

[ref-12] Castro ME, Molina R, Díaz W, Pichuantes SE, Vásquez CC (2008). The dihydrolipoamide dehydrogenase of *Aeromonas caviae* ST exhibits NADH dependent tellurite reductase activity. Biochemical and Biophysical Research Communications.

[ref-13] Chasteen TG, Bentley R (2003). Biomethylation of selenium and tellurium: microorganisms and plants. Chemical Reviews.

[ref-14] Chasteen TG, Fuentes DE, Tantaleán JC, Vásquez CC (2009). Tellurite: history, oxidative stress, and molecular mechanisms of resistance. FEMS Microbiology Reviews.

[ref-15] Che S, Song L, Song W, Yang M, Liu G, Lin X (2013). Complete genome sequence of antarctic bacterium *Psychrobacter* sp. strain G. Genome Announcements.

[ref-16] Cock P, Antao T, Chang J, Chapman B, Cox C, Dalke A, Friedberg I, Hamelryck T, Kauff F, Wilczynski B, De Hoon MJ (2009). Biopython: freely available Python tools for computational molecular biology and bioinformatics. Bioinformatics.

[ref-17] D’Amico S, Colling T, Marx JC, Felle G, Gerdar C (2006). Psychrophilic microorganisms: challenges for life. EMBO Reports.

[ref-18] De Souza MJ, Nair S, Loka Bharathi PA, Chandramohan D (2006). Metal and antibiotic-resistance in psychrotrophic bacteria from Antarctic marine waters. Ecotoxicology.

[ref-19] Deckers M, Balleininger M, Vukotic M, Römpler K, Bareth B, Juris L, Dudek J (2014). Aim24 stabilizes respiratory chain supercomplexes and is required for efficient respiration. FEBS Letters.

[ref-20] Denner EBM, Mark B, Busse HJ, Turkiewicz M, Lubitz W (2001). *Psychrobacter proteolyticus* sp. nov., a psychrotrophic, halotolerant bacterium isolated from the Antarctic krill *Euphausia superba* Dana, excreting a cold-adapted metalloprotease. Systematic and Applied Microbiology.

[ref-21] Dziewit L, Bartosik D (2014). Plamids of psychrophilic and psychrotolerant bacteria and their role in adaptation to cold environments. Frontiers in Microbiology.

[ref-22] Edgar RC (2004). MUSCLE: multiple sequence alignment with high accuracy and high throughput. Nucleic Acids Research.

[ref-23] Elías AO, Abarca MJ, Montes RA, Chasteen TG, Pérez-Donoso JM, Vásquez CC (2012). Tellurite enters *Escherichia coli* mainly through the PitA phosphate transporter. MicrobiologyOpen.

[ref-24] Fani R, Brilli M, Liò P (2005). The origin and evolution of operons: the piecewise building of the proteobacterial histidine operon. Journal of Molecular Evolution.

[ref-25] Felsenstein J (1985). Confidence limits on phylogenies: an approach using the bootstrap. Evolution.

[ref-26] Fourment M, Holmes EC (2016). Seqotron: a user-friendly sequence editor for Mac OS X. BMC Research Notes.

[ref-27] Hu Y, Yan C, Hsu C, Chen Q, Niu K, Komatsoulis G, Meerzaman D (2014). OmicCircos: a simple-to-use R package for the circular visualization of multidimensional omics data. Cancer Informatics.

[ref-28] Juhnke S, Peitzsch N, Hübener N, Große C, Nies DH (2002). New genes involved in chromate resistance in *Ralstonia metallidurans* strain CH34. Archives of Microbiology.

[ref-29] Juni E, Heym GA (1986). *Psychrobacter immobilis* gen. nov., sp. nov.: genospecies composed of Gram-negative, aerobic, oxidase-positive coccobacilli. International Journal of Systematic Bacteriology.

[ref-30] Kämpfer P, Kroppenstedt RM (1996). Numerical analysis of fatty acid patterns of coryneform bacteria and related taxa. Canadian Journal of Microbiology.

[ref-31] Kudo T, Kidera A, Kida M, Kawauchi A, Shimizu R, Nakahara T, Zhang X, Yamada A, Amano M, Hamada Y, Taniyama S, Arakawa O, Yoshida A, Oshima K, Suda W, Kuwahara H, Nogi Y, Kitamura K, Yuki M, Iida T, Moriya S, Inoue T, Hongoh Y, Hattori M, Ohkuma M (2014). Draft genome sequences of *Psychrobacter* strains JCM 18900, JCM 18901, JCM 18902, and JCM 18903, isolated preferentially from frozen aquatic organisms. Genome Announcements.

[ref-32] Lane DJ, Stackebrandt E, Goodfellow M (1991). 16S/23S rRNA sequencing. Nucleic acid techniques in bacterial systematics.

[ref-33] Lanfear R, Frandsen PB, Wright AM, Senfeld T, Calcott B (2016). PartitionFinder 2: new methods for selecting partitioned models of evolution for molecular and morphological phylogenetic analyses. Molecular Biology and Evolution.

[ref-34] Lasa A, Romalde JL (2017). Genome sequence of three *Psychrobacter* sp. strains with potential applications in bioremediation. Genomics Data.

[ref-35] Lemire JA, Harrison JJ, Turner RJ (2013). Antimicrobial activity of metals: mechanisms, molecular targets and applications. Nature Reviews Microbiology.

[ref-36] Lo Giudice A, Casella P, Bruni V, Michaud L (2013). Response of bacterial isolates from Antarctic shallow sediments towards heavy metals, antibiotics and polychlorinates biphenyls. Ecotoxicology.

[ref-37] Makarova KS, Aravind L, Wolf YI, Tatusov RL, Minton KW, Koonin EV, Daly MJ (2001). Genome of the extremely radiation-resistant bacterium *Deinococcus radiodurans* viewed from the perspective of comparative genomics. Microbiology and Molecular Biology Reviews.

[ref-38] Mata MT, Baquero F, Pérez-Díaz JC (2000). A multidrug efflux transporter in *Listeria monocytogenes*. FEMS Microbiology Letters.

[ref-39] Miranda AT, González MV, González G, Vargas E, Campos-García J, Cervantes C (2005). Involvement of DNA helicases in chromate resistance by *Pseudomonas aeruginosa* PAO1. Mutation Research.

[ref-40] Moghadam MS, Albersmeier A, Winkler A, Cimmino L, Rise K, Hohmann-Marriot MF, Kalinowski J, Rückert C, Wentzel A, Lale R (2016). Isolation and genome sequencing of four Arctic marine *Psychrobacter* strains exhibiting multicopper oxidase activity. BMC Genomics.

[ref-41] Morgan M, Pagès H, Obenchain V, Hayden N (2016). http://bioconductor.org/packages/release/bioc/html/Rsamtools.html.

[ref-42] Moscoso H, Saavedra C, Loyola C, Pichuantes S, Vásquez CC (1998). Biochemical characterization of tellurite-reducing activities of *Bacillus stearothermophilus* V. Research in Microbiology.

[ref-43] Ni B, Zhang Y, Huang X, Yang R, Zhou D (2014). Transcriptional regulation mechanism of *ter* operon by OxyR in *Yersinia pestis*. Current Microbiology.

[ref-44] O’Gara JP, Gomelsky M, Kaplan S (1997). Identification and molecular genetic analysis of multiple loci contributing to high-level tellurite resistance in *Rhodobacter sphaeroides* 2.4.1. Applied and Environmental Microbiology.

[ref-45] Pal C, Bengtsson-Palme J, Rensing C, Kristiansson E, Joakim Larsson DG (2014). BacMet: antibacterial biocide and metal resistance genes database. Nucleic Acids Research.

[ref-46] Park CH, Keyhan M, Wielinga B, Fendorf S, Matin A (2000). Purification to homogeneity and characterization of a novel *Pseudomonas putida* chromate reductase. Applied and Environmental Microbiology.

[ref-47] Pérez JM, Calderón IL, Arenas FA, Fuentes DE, Pradenas GA, Fuentes EL, Sandoval JM, Castro ME, Elías AO, Vásquez CC (2007). Bacterial toxicity of potassium tellurite: unveiling an ancient enigma. PLOS ONE.

[ref-48] Potts M (1994). Dessication tolerance of prokaryotes. Microbiological Reviews.

[ref-49] Prigent-Combaret C, Sanguin H, Champier L, Bertrand C, Monnez C, Colinon C, Blaha D, Ghigo JM, Cournoyer B (2012). The bacterial thiopurine methyltransferase tellurite resistance process is highly dependent upon aggregation properties and oxidative stress response. Environmental Microbiology.

[ref-50] Pritchard L, Glover RH, Humphris S, Elphinstone JG, Toth IK (2016). Genomics and taxonomy in diagnostics for food security: soft-rotting enterobacterial plant pathogens. Analytical Methods.

[ref-51] Pugin B, Cornejo F, Muñoz Díaz P, Muñoz Villagrán C, Vargas-Pérez J, Arenas FA, Vásquez CC (2014). Glutathione reductase-mediated synthesis of tellurium-containing nanostructures exhibiting antibacterial properties. Applied and Environmental Microbiology.

[ref-52] R Development Core Team (2011).

[ref-53] Rangannan V, Bansal M (2010). High-quality annotation of promoter regions for 913 bacterial genomes. Bioinformatics.

[ref-54] Rensing C, Grass G (2003). *Escherichia coli* mechanism of copper homeostasis in a changing environment. FEMS Microbiology Reviews.

[ref-55] Rodríguez-Rojas F, Díaz-Vásquez W, Undabarrena A, Muñoz Díaz P, Arenas F, Vásquez C (2016). Mercury-mediated cross-resistant to tellurite in *Pseudomonas spp* isolated from the Chilean Antarctic territory. Metallomics.

[ref-56] Romanenko LA, Schumann P, Rohde M, Lysenko AM, Mikhailov V, Stackebrandt E (2002). *Psychrobacter submarinus* sp and *Psychrobacter marincola* sp. nov., psychrophilic halophiles from marine environments. International Journal of Systematic and Evolutionary Microbiology.

[ref-57] Ronquist F, Teslenko M, Van der Mark P, Ayres DL, Darling A, Höhna S, Larget B, Liu L, Suchard MA, Huelsenbck JP (2012). MrBayes 3.2: efficient Bayesian phylogenetic inference and model choice across a large model space. Systematic Biology.

[ref-58] Salzberg SL, Deicher AL, Kasif S, White O (1998). Microbial gene identification using interpolated Markov models. Nucleic Acids Research.

[ref-59] Sambrook JR, Russell DW (2001). Molecular cloning: a laboratory manual.

[ref-60] Sanssouci E, Lerat S, Grondin G, Shareck F, Beaulieu C (2011). Tdd8: a TerD domain-encoding gene involved in *Streptomyces coelicolor* differentiation. Antonie Van Leeuwenhoek.

[ref-61] Seemann T (2014). Prokka: rapid prokaryotic genome annotation. Bioinformatics.

[ref-62] Simão FA, Waterhouse RM, Ioannidis P, Kriventseva EV, Zdobnov EM (2015). BUSCO: assessing genome assembly and annotation completeness with single-copy orthologs. Bioinformatics.

[ref-63] Tamura K, Stecher G, Peterson D, Filipski A, Kumar S (2013). MEGA6: molecular evolutionary genetics analysis version 6.0. Molecular Biology and Evolution.

[ref-64] Tanenbaum DM, Goll J, Murphy S, Kumar P, Zafar N, Thiagarajan M, Madupu R, Davidsen T, Kagan L, Kravitz S, Rusch DB, Yooseph S (2010). The JCVI standard operating procedure for annotating prokaryotic metagenomic shotgun sequencing data. Standards in Genomic Sciences.

[ref-65] Taylor DE (1999). Bacterial tellurite resistance. Trends in Microbiology.

[ref-66] Taylor DE, Rooker M, Keelan M, Ng LK, Martin I, Perna NT, Burland NT, Blattner FR (2002). Genomic variability of O islands encoding tellurite resistance in enterohemorrhagic *Escherichia coli* O157: H7 isolates. Journal of Bacteriology.

[ref-67] Taylor DE, Walter EG, Sherburne R, Bazett-Jones DP (1988). Structure and location of tellurium deposited in *Escherichia coli* harbouring tellurite resistance plasmids. Journal Ultrastructure and Molecular Structure Research.

[ref-68] Toptchieva A, Sisson G, Bryden LJ, Taylor DE, Hoffman PS (2003). An inducible tellurite-resistance operon in *Proteus mirabilis*. Microbiology.

[ref-69] Tritt A, Eisen JA, Facciotti MT, Darling AE (2012). An integrated pipeline for *de novo* assembly of microbial genomes. PLOS ONE.

[ref-70] Turkovicova L, Smidak R, Jung G, Turna J, Lubec G, Aradska J (2016). Proteomic analysis of the TerC interactome: novel links to tellurite resistance and pathogenicity. Journal of Proteomics.

[ref-71] Turner RJ, Borghese R, Zannoni D (2012). Microbial processing of tellurium as a tool in biotechnology. Biotechnology Advances.

[ref-72] Turner S, Pryer KM, Miao VP, Palmer JD (1999). Investigating deep phylogenetic relationships among cyanobacteria and plastids by small submit rRNA sequence analysis. Journal of Eukaryotic Microbiology.

[ref-73] Varghese NJ, Mukherjee S, Ivanova N, Konstantinidis KT, Mavrommatis K, Kyrpides NC, Pati A (2015). Microbial species delineation using whole genome sequences. Nucleic Acids Research.

[ref-74] Whelan KF, Colleran E (1992). Restriction endonuclease mapping of the HI2 incompatibility group plasmid R478. Journal of Bacteriology.

[ref-75] Whelan KF, Colleran E, Taylor DE (1995). Phage inhibition, colicin resistance, and tellurite resistance are encoded by a single cluster of genes on the IncHI2 plasmid R478. Journal of Bacteriology.

[ref-76] Whelan KF, Sherburne RK, Taylor DE (1997). Characterization of a region of the IncHI2 plasmid R478 which protects *Escherichia coli* from toxic effects specified by components of tellurite, phage and colicin resistance cluster. Journal of Bacteriology.

[ref-77] Wickham H (2007). Reshaping data with reshape package. Journal of Statistical Software.

[ref-78] Wu M, Eisen JA (2008). A simple, fast, and accurate method of phylogenetic inference. Genome Biology.

