# Supplementary figures and images for: Comparative genomic analysis of a new tellurite-resistant Psychrobacter strain isolated from the Antarctic Peninsula
Source: PeerJ. 2018 Feb 19;6:e4402. doi: 10.7717/peerj.4402 (PMC5822837; doi:10.7717/peerj.4402)

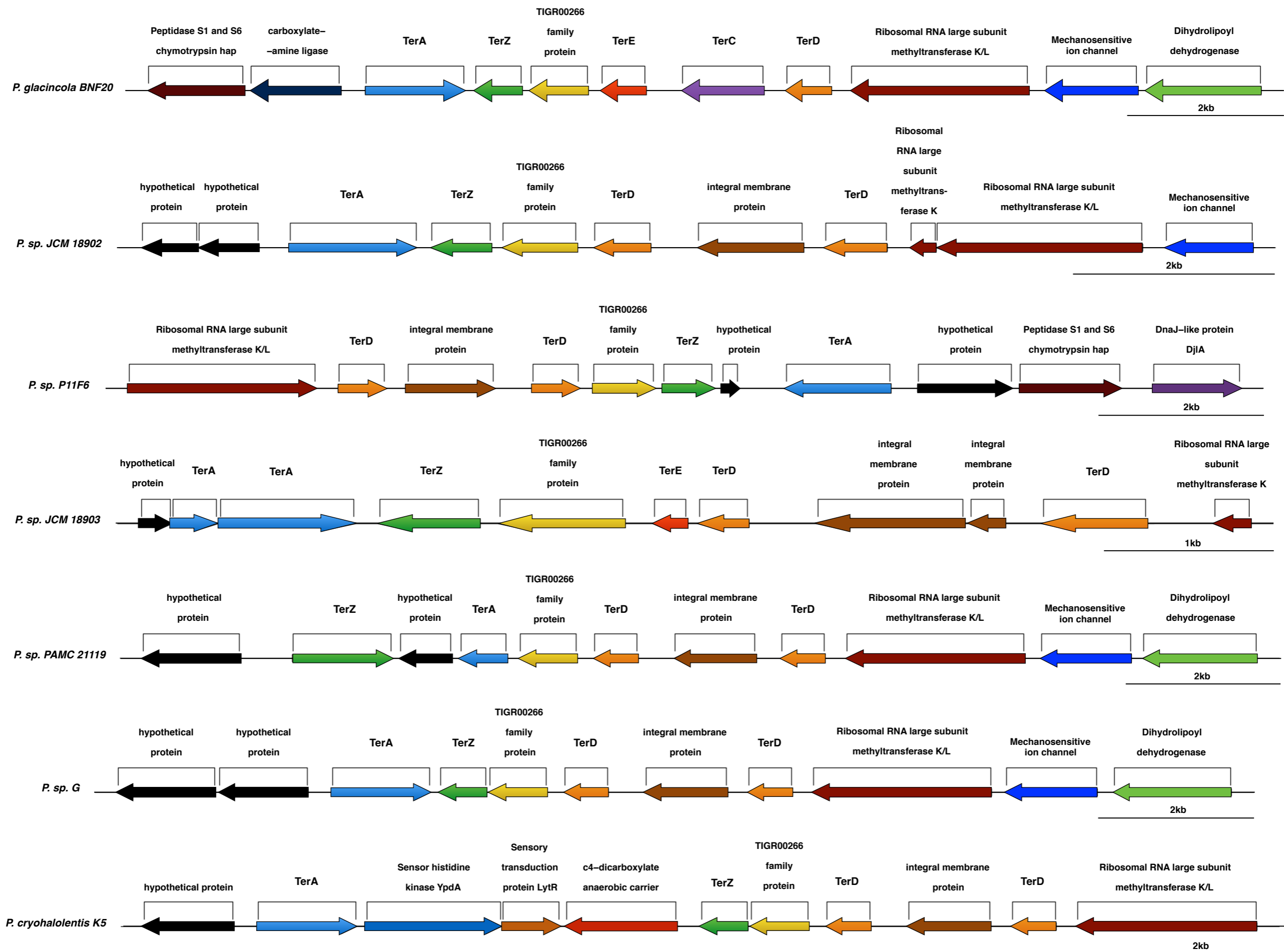

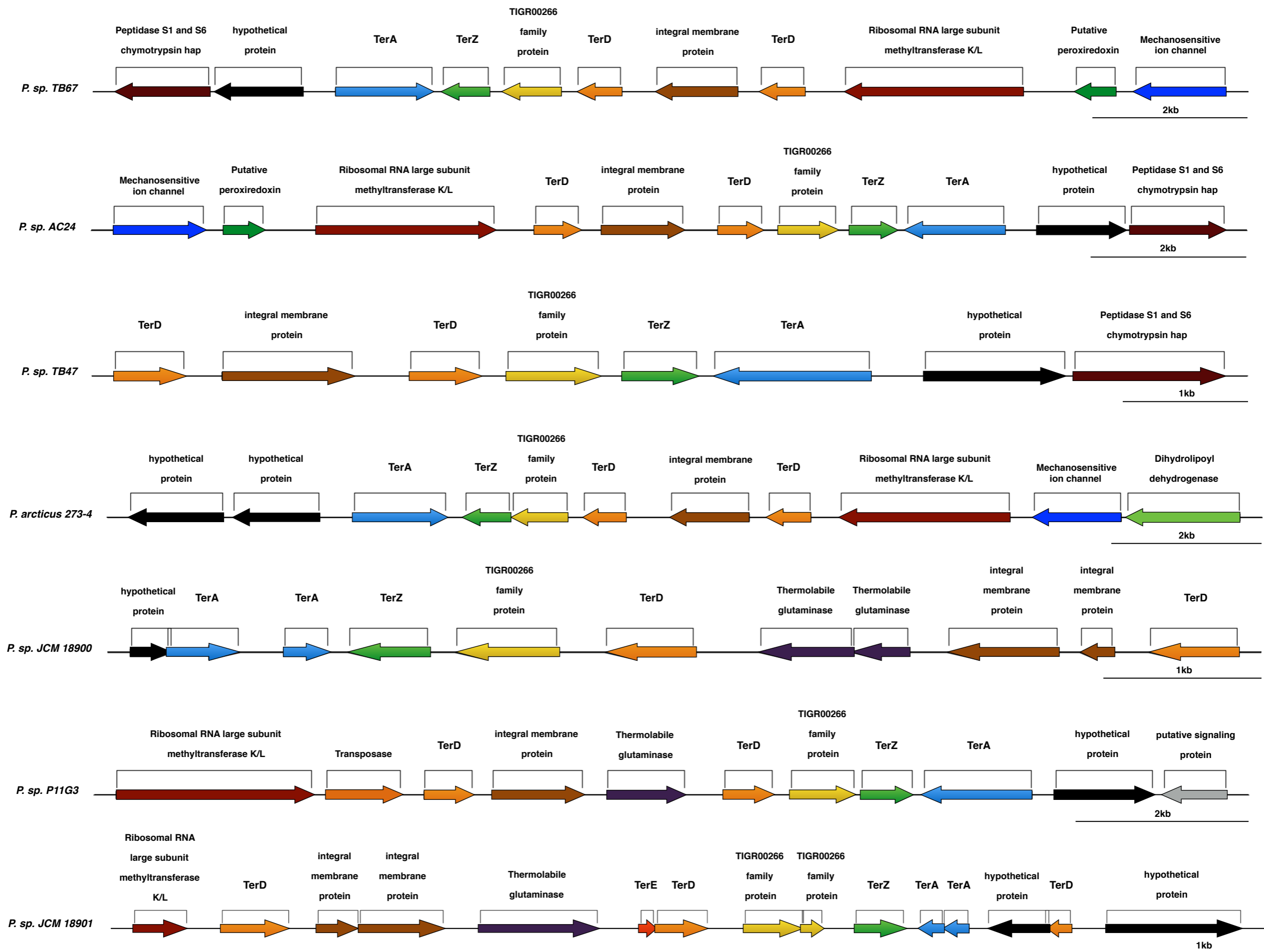

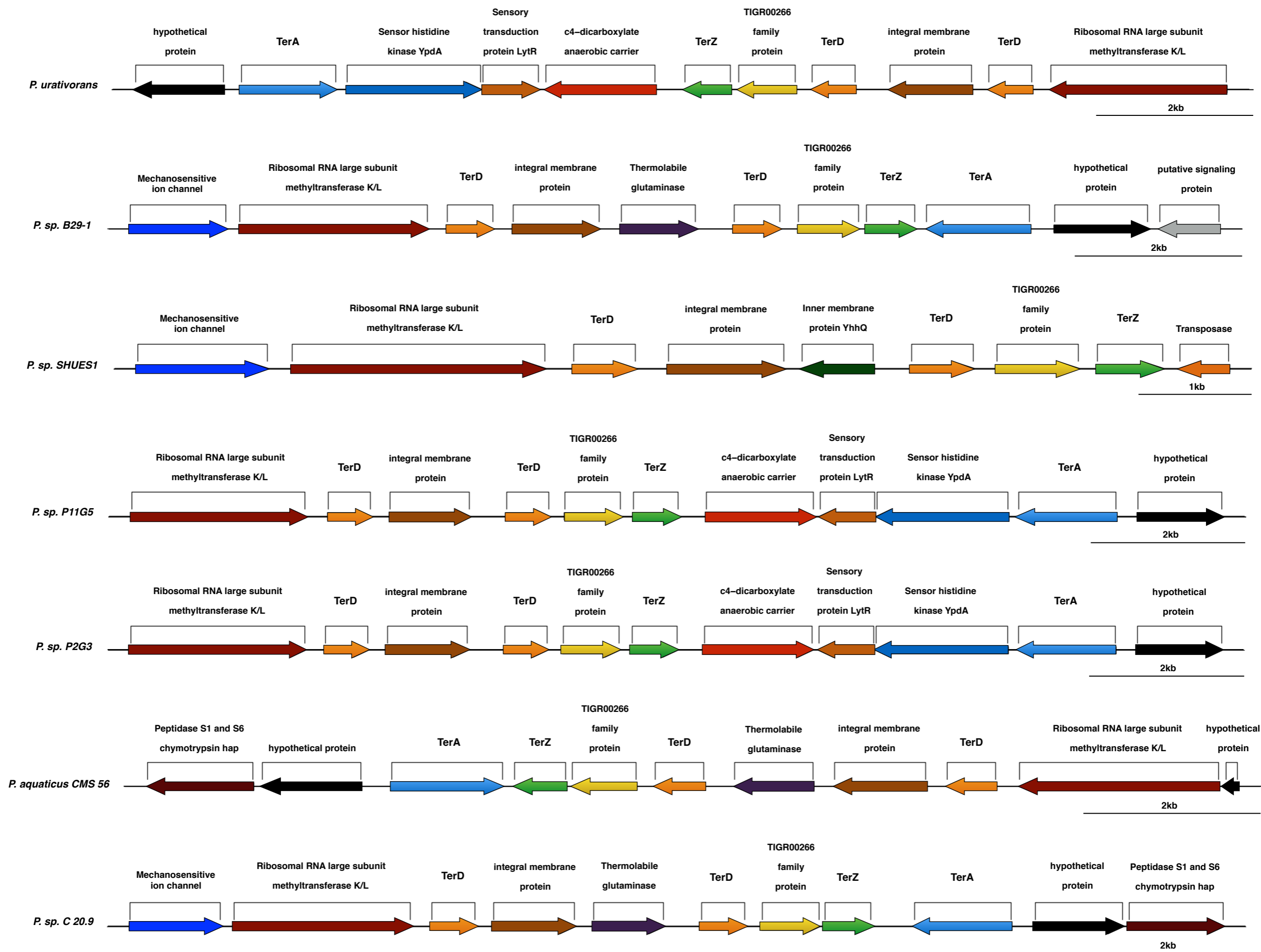

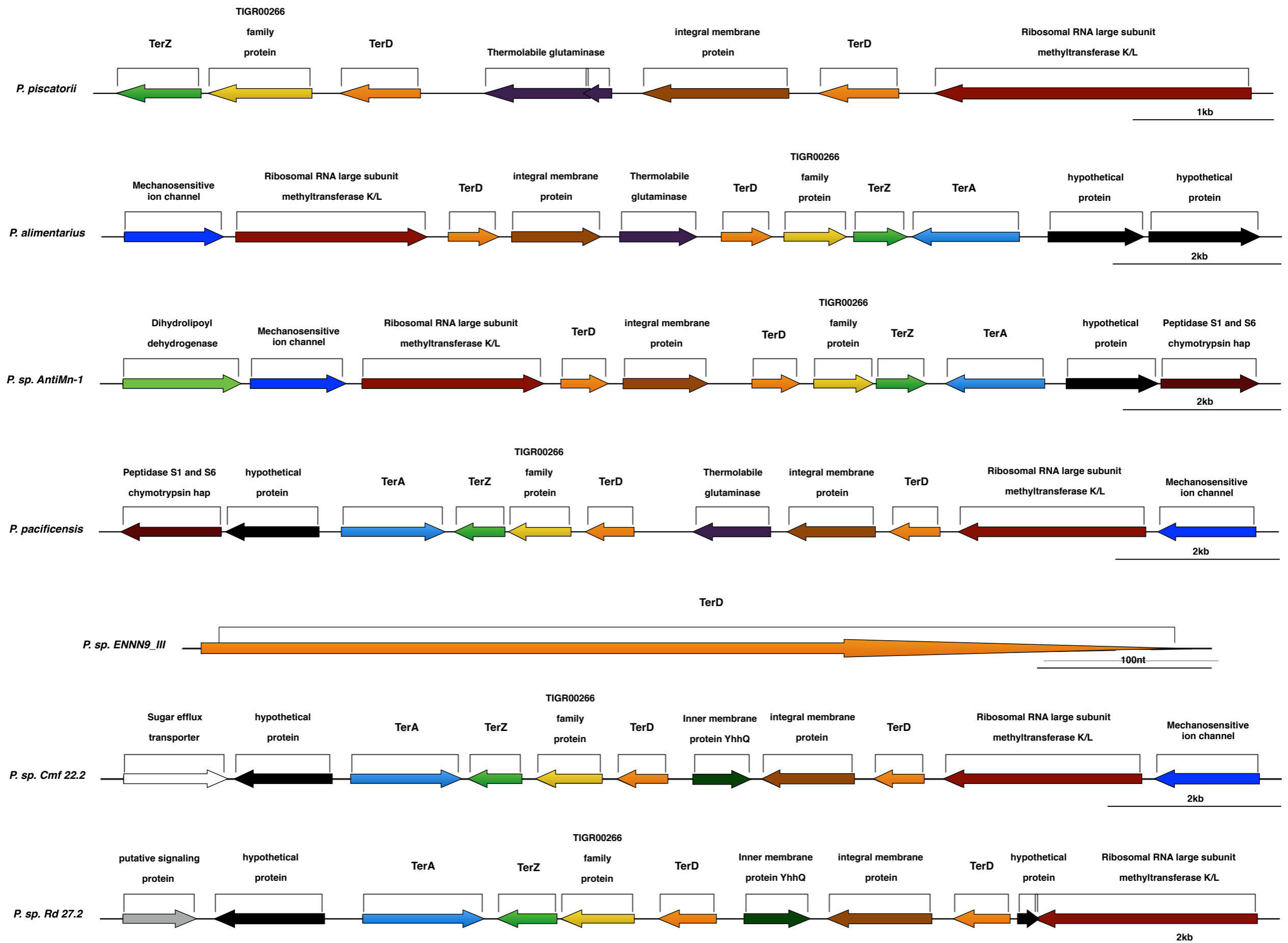

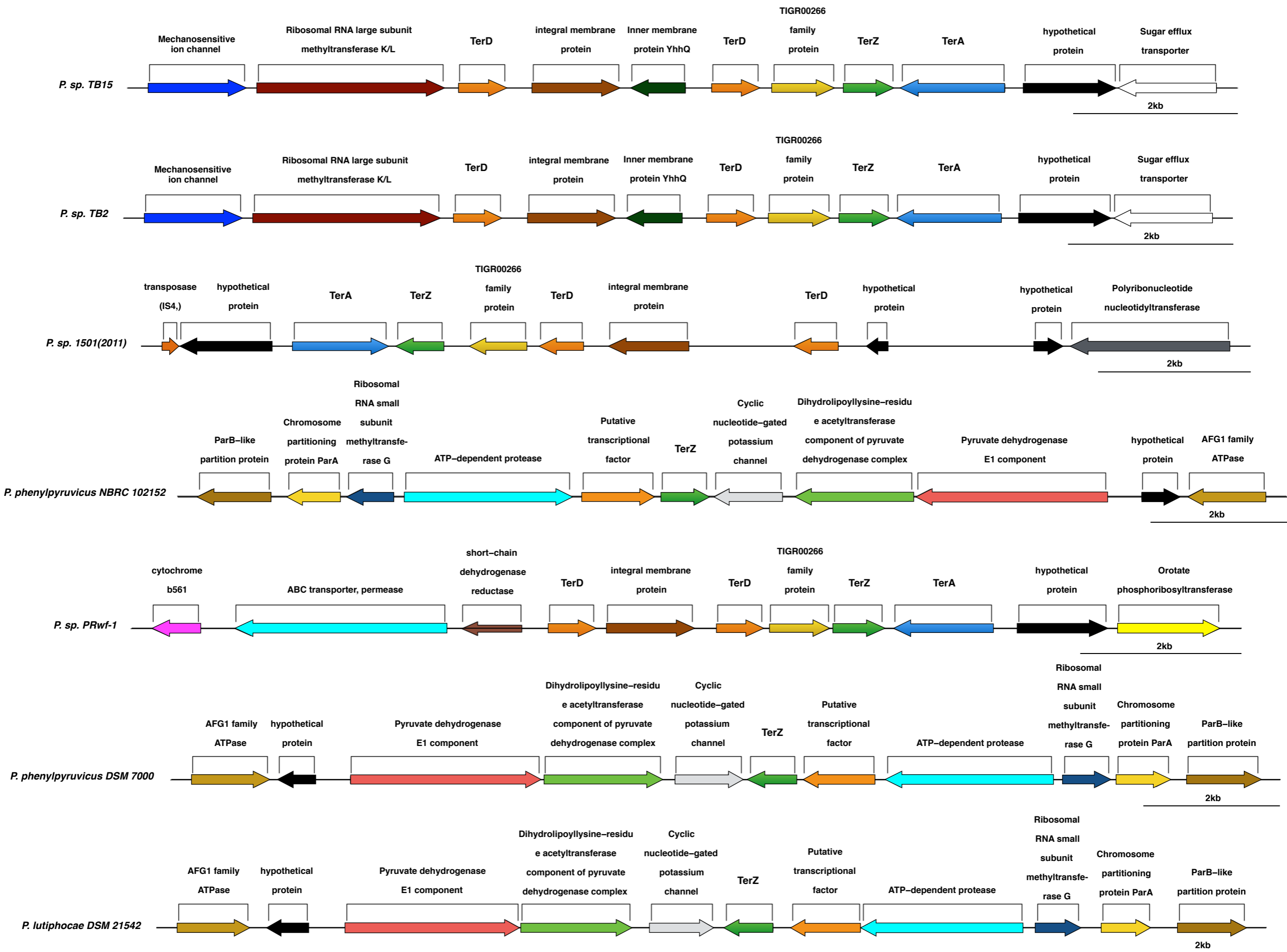

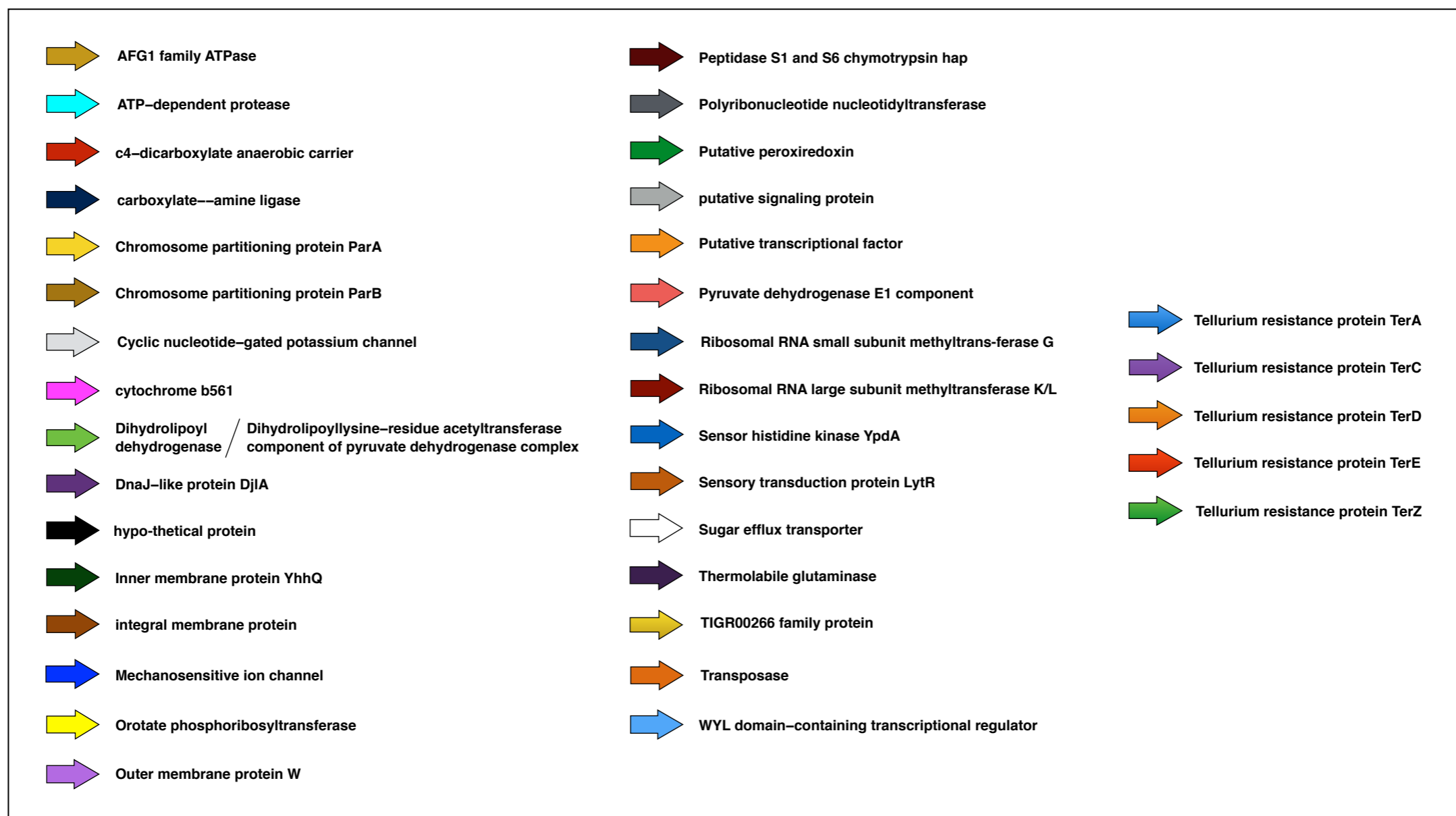

**Figure S1.** Genetic context of *ter* genes in the indicated *Psychrobacter* species.

Supplement: Figure S1 [file peerj-06-4402-s005.pdf]
